# A Phase 3 Multicenter, Double‐Blind Study Comparing Efficacy, Safety, Immunogenicity, and Pharmacokinetics of Alkem's Biosimilar Teriparatide Versus Reference Teriparatide in Postmenopausal Osteoporosis

**DOI:** 10.1002/agm2.70029

**Published:** 2025-06-13

**Authors:** Nitin Kapoor, Thomas Paul, Rajeshwar Nath Srivastava, Saurabh Singh, Sunil Maheshwari, Vishal Patil, Awadhesh Kumar Yadav, Girish Bhatia, Sushil H. Mankar, Joe Joseph Cherian, Surabhi Maheshwari, Sudeepti Srivastava, Dattatray Pawar, Roshan Pawar, Amol Aiwale, Amitrajit Pal, Yogesh Rane, Vinayaka Shahavi, Akhilesh Sharma

**Affiliations:** ^1^ Christian Medical College Vellore India; ^2^ King George Medical University Lucknow India; ^3^ Banaras Hindu University Varanasi India; ^4^ Medilink Hospital and Research Centre Ahmedabad India; ^5^ Lifepoint Multispecialty Hospital Pune India; ^6^ Om Surgical Center & Maternity Home Varanasi India; ^7^ Medipoint Hospitals Pvt. Ltd. Pune India; ^8^ NKPSIMS and Lata Mangeshkar Hospital Nagpur India; ^9^ St. Johns Medical College Hospital Bangalore India; ^10^ Medical Affairs Department Alkem Laboratories Ltd Mumbai India; ^11^ Clinical Research Department Alkem Laboratories Ltd Mumbai India

**Keywords:** biosimilar, bone mineral density, PN1P, postmenopausal osteoporosis, teriparatide

## Abstract

**Objective:**

The primary purpose of this study was to compare the efficacy and safety of proposed biosimilar teriparatide with reference teriparatide in patients of postmenopausal osteoporosis. The secondary objectives were to assess the pharmacodynamic response of study drugs in postmenopausal osteoporosis and to assess the pharmacokinetic profile of biosimilar and reference teriparatide in a subset of subjects (a total of 30 evaluable subjects i.e., 15 subjects in reference arm and 15 subjects in biosimilar arm).

**Methods:**

A prospective, active‐controlled, randomized, double‐blind, phase III study included postmenopausal women (50–80 years of age) with at least 5 years since menopause diagnosed with osteoporosis (*T‐SCORE* ≤ −2.5 SD at lumbar spine or femoral neck) randomized 2:1 to receive either Alkem's biosimilar teriparatide or reference teriparatide 20 μg once daily subcutaneously for 48 weeks. All subjects received calcium 1000 mg and vitamin D3 500 IU once daily orally. The primary efficacy endpoint was percent change in bone mineral density (BMD) at lumbar spine and femoral neck from baseline to 48 weeks. Safety outcomes, pharmacokinetics, and immunogenicity were also evaluated. Secondary endpoints included change from baseline in pharmacodynamic parameters like serum P1NP, which were analyzed at randomization, at week 12, 24, and 48.

**Results:**

In total, 177 patients (114 in biosimilar group and 63 in reference group) were randomized. The percent change from baseline to 48 weeks in lumbar spine BMD (least square mean [LSM] ± standard error [SE]) was 8.58% ± 0.85 in the biosimilar group and 8.02% ± 1.23 in the reference group. The estimated between‐group difference (95% confidence interval [CI]) was −0.56% (−2.43% to 3.54%) within the prespecified noninferiority margin (− 2.43%), which indicates noninferiority of biosimilar teriparatide compared to reference teriparatide. The percent change in femoral neck BMD from baseline to 48 weeks (LSM ± SE) was 3.94% ± 0.83 in the biosimilar group and 2.50% ± 1.20 in the reference group. The estimated between‐group difference (95% CI) was 1.44% (−1.44% to 4.32%) within the prespecified noninferiority margin (−1.44%) indicating noninferiority of biosimilar teriparatide compared to reference teriparatide. Changes in P1NP (serum procollagen type 1 N terminal pro‐peptide) were also similar between the groups. Safety profiles, including immunogenicity, were comparable.

**Conclusion:**

This study established noninferiority, along with comparable safety and immunogenicity between Alkem's biosimilar teriparatide and reference teriparatide in patients with postmenopausal osteoporosis.

**Trial Registration:**

CTRI number: CTRI/2018/05/014254

## Graphical abstract

This phase III study demonstrated that Alkem's biosimilar teriparatide is noninferior to reference teriparatide in improving bone mineral density at the lumbar spine and femoral neck in postmenopausal osteoporosis patients, with comparable safety, pharmacodynamics, and immunogenicity profiles over 48 weeks of treatment.

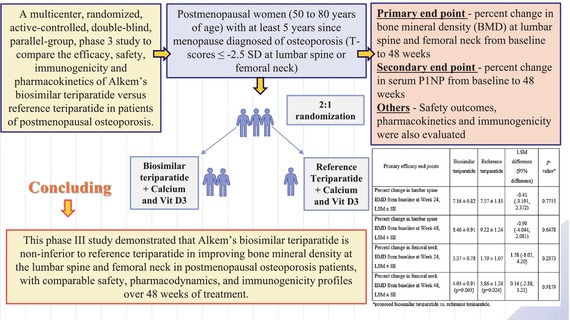

## 1 Introduction

Osteoporosis is a significant global health concern, particularly among postmenopausal women, with an estimated 40%–50% experiencing fragility fractures during their lifetime [1, 2, 3]. These fractures are associated with considerable morbidity and mortality. The primary objective of osteoporosis management is to reduce the risk of fractures, given their prolonged healing process. Preventive strategies include supplementation with calcium and vitamin D, fall prevention, smoking cessation, and moderated alcohol consumption. These measures are particularly crucial for postmenopausal women, focusing on minimizing bone loss [4, 5, 6, 7].

The administration of parathyroid hormone (PTH) has been demonstrated to enhance bone mineral density (BMD) in estrogen‐deficient women, thereby reducing the risk of fractures in those with postmenopausal osteoporosis [4, 5, 6, 7]. Unlike other treatments that primarily inhibit bone resorption, PTH stimulates bone formation. Teriparatide, a recombinant form of PTH, represents the first potent anabolic agent approved for the treatment of osteoporosis [8, 9]. By promoting osteoblast function, differentiation, and survival, teriparatide facilitates new bone formation and has been shown to significantly lower fracture risk [10, 11, 12, 13]. It is indicated for severe osteoporosis and is administered as a once daily subcutaneous injection at a dose of 20 μg [14, 15, 16, 17].

The present study aims to evaluate the equivalence in efficacy and safety between biosimilar and reference teriparatide formulations for the treatment of postmenopausal osteoporosis.

In India, osteoporosis constitutes a major public health challenge, particularly among postmenopausal women. Contributing factors include poor dietary calcium intake, widespread vitamin D deficiency, and lifestyle changes, particularly in urban populations. The economic burden of osteoporosis is substantial, encompassing both direct medical costs and indirect losses due to disability and reduced productivity. Effective management of the condition is hindered by low awareness, insufficient screening, and limited access to treatment options [18, 19].

Although teriparatide is effective, its high cost poses a significant barrier for many Indian patients. The availability of biosimilar teriparatide provides a more cost‐effective alternative, offering comparable safety and efficacy. These biosimilars, approved for use in India, enhance competition in the pharmaceutical market, thereby lowering treatment costs. This improved affordability increases patient access to effective therapy and alleviates the economic burden of osteoporosis on individuals and healthcare systems [20].

## 2 Methods

### 2.1 Study Design and Patient Selection

**FIGURE 1:**
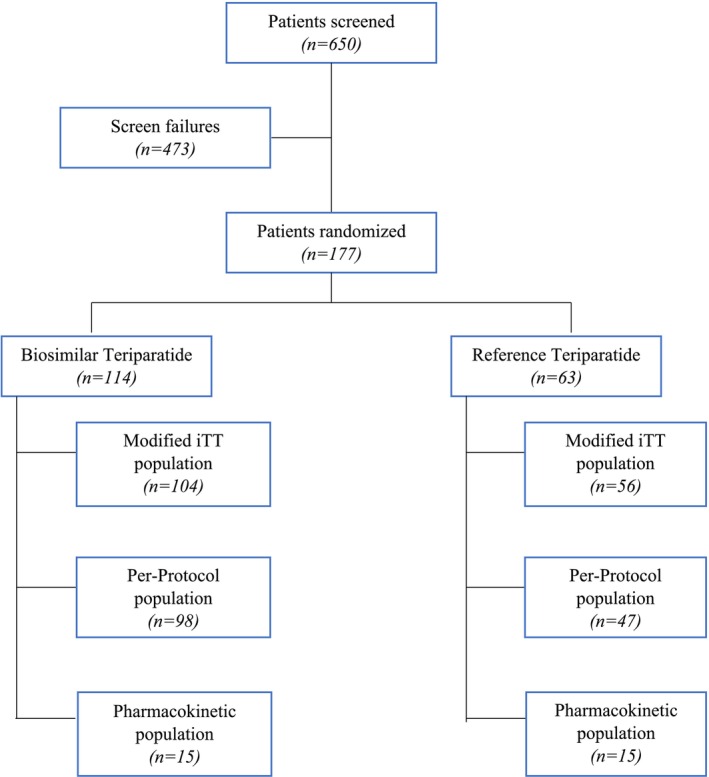
Patient disposition.

A multicenter, randomized, active‐controlled, double‐blind, parallel‐group phase III study was conducted between August 2018 and August 2020 at 17 sites in India. The study was conducted as per the principles and requirements of the Declaration of Helsinki (2013), and ICH‐GCP (E6‐R2, Step 4) guidelines along with the local regulatory requirements of Good Clinical Practices for Clinical Research in India (2004), ICMR guidelines for Biomedical Research on Human Subjects (2017) and New Drugs and Clinical Trial Rules, 2019 (CDSCO) and after approvals from respective Institutional Ethics Committees. The study was registered on the Clinical Trial Registry of India (CTRI/2018/05/014254). Figure 1 shows the patient disposition.

### 2.2 Determination of Sample Size

Considering the mean difference in lumber spine BMD of 6%, with a common standard deviation of 8% and a noninferiority limit of 10%, 67 subjects per arm have been estimated to have 90% power at a 5% level of significance.

In order to make allocation of 2:1 sample size, the following formulae were used:

$$n_{1}=\left(0.5\right)\times n\times \left(1+k\right)$$

$$n_{2}=\left(0.5\right)\times n\times \left(1+\left(1/k\right)\right)$$

With these assumptions, 100 subjects are estimated to enroll in the test arm and 50 for the reference arm. Considering a 15% dropout rate, the total sample size of 177 randomized subjects has been proposed for this study (118 for test arm and 59 for reference arm to randomize).

After signing of the informed consent form, the subjects were screened to assess eligibility according to the study selection criteria. Postmenopausal women between the ages of 50–80 years with BMD *T‐SCORE* less than −2.5 at the lumbar spine or femoral neck were enrolled in the study.

Women were excluded from the study if they had conditions influencing bone metabolism, such as primary hyperparathyroidism, thyroid disorders, hypercalcemia, or vitamin D deficiency. Exclusion criteria also included prior use of bisphosphonates within the past 12 months or treatment with any medication affecting bone metabolism within the last 3 months. Additionally, women with a history of hip replacement surgery, vertebral abnormalities from L1 to L4 that could interfere with vertebral assessment via dual‐energy X‐ray absorptiometry (DXA), or evidence of metabolic bone diseases other than osteoporosis, such as Paget's disease, were excluded.

The trial and consent process was approved by the institutional review boards and ethics committees overseeing the various study sites.

### 2.3 Treatment Plan

During the treatment phase, following a screening period of 3 weeks, eligible subjects were randomly assigned in a 2:1 ratio to receive either biosimilar teriparatide (Alkem Laboratories Ltd., Mumbai, or Enzene Biosciences Ltd., Pune) or reference teriparatide (Eli Lilly and Company India Pvt. Ltd.). Both groups received 20 μg of teriparatide via subcutaneous self‐injection once daily for a treatment duration of 48 weeks, followed by a 2‐week follow‐up period. All participants were provided with daily oral supplementation of 1000 mg of calcium and 500 IU of vitamin D3.

Medications known to affect bone metabolism, such as bisphosphonates, raloxifene, and denosumab, as well as drugs associated with reduced bone density, including glucocorticoids, aromatase inhibitors, and tamoxifen, were prohibited during the study. Throughout the treatment period, data were collected for primary and secondary endpoints. BMD was evaluated using dual‐energy X‐ray absorptiometry (DXA) as part of the efficacy assessments. Laboratory samples were collected during the treatment phase to support efficacy and safety evaluations, and all laboratory analyses were conducted at a central laboratory.

Pharmacokinetic (PK) assessments were performed on a subset of subjects who voluntarily consented to participate in an additional PK sub‐study alongside the main study. A total of 30 evaluable subjects (15 in the biosimilar teriparatide arm and 15 in the reference teriparatide arm) participated in the PK sub‐study. Blood samples for PK analysis were collected at the study site on Day 1 (Visit 2, i.e., the randomization visit) at specified time points: pre‐dose (within 30 min before the first investigational product [IP] administration) and at 5‐, 10‐, 20‐, 30‐, 45‐, 60‐, 90‐, 120‐, 180‐, and 240‐min post‐IP administration. Plasma concentrations of teriparatide were analyzed for both the test and reference products, and PK parameters, such as maximum concentration (*C*_max_), area under the curve from time 0 to the last measurable concentration (AUC_0–*t*_), and time to maximum concentration (*T*_max_) were assessed.

Following the conclusion of the treatment phase, a posttreatment follow‐up visit was conducted for all subjects 14 ± 3 days after the final treatment visit to evaluate the overall clinical outcomes.

### 2.4 Efficacy and Safety Variables

The primary endpoint of the study was the percent change in BMD of the lumbar spine and proximal femur, assessed using dual‐energy X‐ray absorptiometry (DXA) from baseline to Weeks 24 and 48. All DXA measurements were analyzed centrally, and the results were not disclosed to individual study centers to ensure unbiased interpretation. Secondary endpoints included the change from baseline in the pharmacodynamic parameter serum P1NP (procollagen type 1 N terminal propeptide), which was assessed at randomization and Weeks 12, 24, and 48. Additional assessments included hematology, urinalysis, and anti‐teriparatide antibody testing, which were conducted at baseline, Week 24, and Week 48. Serum biochemistry analyses were performed at baseline and at Weeks 4, 12, 24, and 48.

### 2.5 Statistical Analysis

The efficacy analysis was performed on all the subjects in mITT (modified intention‐to‐treat) and PP (per protocol) population. Any missing postbaseline data were imputed using the last observation carried forward (LOCF) procedure. For the analysis of change from baseline to Week 12 (for P1NP), Week 24, and Week 48, a regression model with percent change from baseline as the response variable and the stratification variable was used for randomization, and treatment as independent variables was fit to test the mean difference between the two treatments in percent change. Adjusted least square mean (LSM) and the standard error values along with the *p* value and the 95% confidence interval (CI) of the difference were presented.

PK parameters (*C*_max_, AUC_0–*t*_, and *T*_max_) measured in plasma were calculated by noncompartmental methods, using Phoenix software for the PK population on Day 1. Descriptive statistics (number of subjects [*n*], mean, standard deviation, median, minimum, and maximum [range]) were calculated for all evaluable PK parameters by treatment group for the PK population.

For safety analyses, all AEs (adverse events) were coded using the Medical Dictionary for Regulatory Activities (MedDRA) and summarized by system organ class.

## 3 Results

### 3.1 Patient Population and Demographic

A total of 650 patients were screened, of which 117 were randomized to either the biosimilar group (biosimilar teriparatide; *n =* 114) or the reference group (reference teriparatide; *n =* 63). A total of 148 patients completed the study. The baseline demographics and characteristics of patients were balanced between the groups (Table 1).

**TABLE 1:** Demographics of both the groups.

Characteristics	Biosimilar teriparatide (*n =* 114)	Reference teriparatide (*n =* 63)	Total (*n =* 177)	*p*
Age in years, mean ± SD	62.70 ± 7.19	61.86 ± 7.42	62.40 ± 7.27	0.461
Height in cm, mean ± SD	149.82 ± 7.40	148.92 ± 7.22	149.50 ± 7.33	0.434
Body weight in kg, mean ± SD	56.19 ± 10.50	55.67 ± 10.58	56.00 ± 10.50	0.754

*Note:* Percentages are based on the number of patients in respective treatment groups in intention‐to‐treat (ITT) population. *p* values are presented using a two‐sample *t*‐test and chi‐square/Fisher exact test between the two groups.

### 3.2 Efficacy

#### 3.2.1 BMD Improvement at Lumbar Spine

There was statistically significant improvement with respect to percentage change from baseline to Week 24 and Week 48 at lumbar BMD in both the treatment groups (*p* < 0.0001; mITT population). There was no significant difference in BMD percentage change between the treatment groups, indicating noninferiority of proposed biosimilar teriparatide to reference teriparatide (Table 2).

**TABLE 2:** Percentage change in BMD at lumbar spine and femoral neck from baseline to Week 24 and Week 48 (mITT population).

Primary efficacy end points	Biosimilar teriparatide	Reference teriparatide	LSM difference (95% difference)	*p* ^a^
Percent change in lumbar spine BMD from baseline at Week 24, LSM **±** SE	7.16 **±** 0.82	7.57 **±** 1.13	−0.410 (−3.191, 2.372)	0.772
Percent change in lumbar spine BMD from baseline at Week 48, LSM **±** SE	8.46 **±** 0.94	9.22 **±** 1.34	−0.754 (−4.011, 2.502)	0.648
Percent change in femoral neck BMD from baseline at Week 24, LSM **±** SE	3.37 ± 0.78	1.79 ± 1.07	1.576 (−1.048, 4.199)	0.237
Percent change in femoral neck BMD from baseline at Week 48, LSM **±** SE	4.03 ± 0.96 (*p* = 0.003)	3.86 ± 1.24 (*p* = 0.024)	0.173 (−3.146, 3.493)	0.918

^a^ Proposed biosimilar teriparatide versus reference teriparatide.

The lower bound of 95% CI (−4.044%) for the difference between the groups in LSM percentage change from baseline to Week 48 in BMD at lumbar spine lay above the noninferiority margin, indicating that the percentage change in lumbar spine BMD at Week 48 in the biosimilar group was noninferior to the reference group.

#### 3.2.2 BMD Improvement at Femoral Neck

**FIGURE 2:**
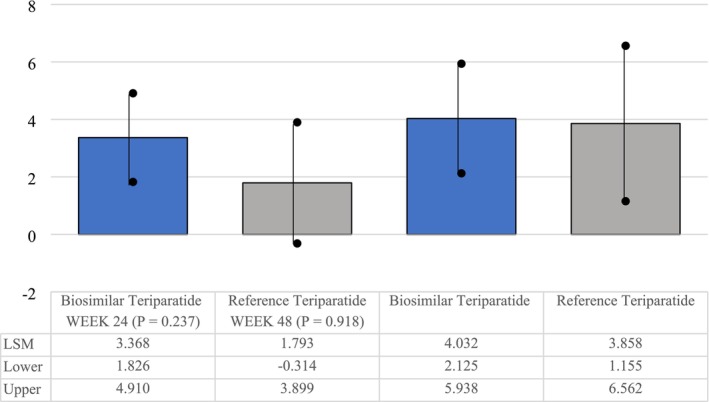
Percentage change (femoral neck—BMD)‐modified intention‐to‐treat (mITT) population.

**FIGURE 3:**
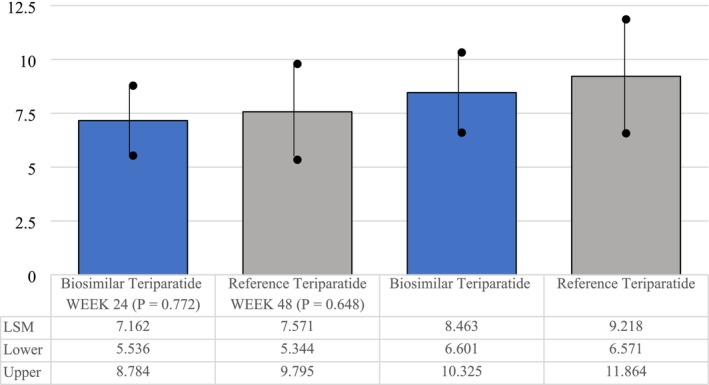
Percentage change (lumber spine—BMD)‐modified intention‐to‐treat (mITT) population.

Similar to lumbar BMD improvements, there was a statistically significant improvement with respect to percentage change from baseline to Week 24 (*p* < 0.0001) and Week 48 (*p* < 0.01) at femoral neck BMD in both the treatment groups (mITT population). There was no significant difference in BMD percentage change between the treatment groups, indicating the noninferiority of the proposed biosimilar teriparatide to reference teriparatide (Table 2; Figures 2 and 3).

The lower bound of 95% CI (−2.883%) for the difference between the groups in LSM percentage change from baseline to Week 48 in BMD at femoral neck lay above the noninferiority margin, indicating that the percentage change in femoral neck BMD at Week 48 in the biosimilar group was noninferior to the reference group.

#### 3.2.3 Improvement in Bone Turnover Marker (P1NP)

There was a statistically significant improvement observed in mean change from baseline to Week 12 (*p* < 0.0001 for biosimilar teriparatide, *p* < 0.001 for reference teriparatide), mean change from baseline to Week 24 (*p* < 0.0001) and mean change from baseline to Week 48 (*p* < 0.0001 for biosimilar teriparatide, *p* < 0.002 for reference teriparatide) in the serum PINP in both treatment groups. There was no statistically significant difference noted in change (improvement) in serum PINP concentration from baseline to endpoint between the two treatment groups at Week 12, Week 24, and Week 48 (Table 3).

**TABLE 3:** Change from baseline in Serum P1NP concentration at Week 12, 24, and 48 (mITT population).

Secondary end points	Biosimilar teriparatide	Reference teriparatide	*p* ^a^
Change from baseline to Week 12 (LSM ± SE)	24.38 ± 4.44	21.95 ± 6.02	0.746
Change from baseline to Week 24 (LSM ± SE)	33.05 ± 7.00	51.07 ± 9.63	0.133
Change from baseline to Week 48 (LSM ± SE)	48.52 ± 7.24	34.30 ± 9.98	0.251

^a^ Proposed biosimilar teriparatide versus reference teriparatide.

### 3.3 Safety

There were no major differences between the treatment groups in the incidence of AEs and serious AEs (serious adverse events). A total of 40.1% (*n* = 71) of patients experienced one or more AEs, most of which were mild or moderate in severity. In the biosimilar group, there were 72 adverse events reported in 36 (31.6%) patients, and 79 adverse events were reported in 35 (55.6%) patients in the reference group. No statistically significant difference (*p* = 0.905) was observed in patients with overall TEAEs (treatment‐emergent adverse events) between the two treatment groups. SAEs were reported in both the biosimilar and reference groups; however, they were not related to either of the study treatments.

The common (i.e., ≥ 1%) TEAEs reported in the proposed biosimilar teriparatide group were: hyperuricaemia (7.0%), pyrexia (5.3%), anemia (2.6%), hypokalemia (1.8%), decreased appetite (1.8%), hyperglycemia (1.8%), gastritis (1.8%), constipation (1.8%), muscle spasms (1.8%), noncardiac chest pain (1.8%), dizziness (1.8%), nasopharyngitis (1.8%), blood pressure increased (1.8%), and hypertension (1.8%).

The common (i.e., ≥ 1%) TEAEs reported in the reference teriparatide group were: pyrexia (7.9%), hyperuricaemia (6.3%), arthralgia (6.3%), pain (6.3%), diarrhea (4.8%), back pain (4.8%), musculoskeletal pain (4.8%), paraesthesia (4.8%), hyponatraemia (3.2%), gastritis (3.2%), toothache (3.2%), pain in extremity (3.2%), asthenia (3.2%), dizziness (3.2%), headache (3.2%), urinary tract infection (3.2%), rash (3.2%), blood pressure increased (3.2%), hypokalaemia (1.6%), hypernatraemia (1.6%), hyperkalaemia (1.6%), hypomagnesaemia (1.6%), aphthous ulcer (1.6%), abdominal pain (1.6%), myalgia (1.6%), hypoaesthesia (1.6%), fungal infection (1.6%), gastroenteritis (1.6%), upper respiratory tract infection (1.6%), anemia (1.6%), radius fracture (1.6%), hypertension (1.6%), pruritus (1.6%), proteinuria (1.6%), and anxiety (1.6%).

### 3.4 Immunogenicity

Anti‐teriparatide antibodies were not detected in any patient in both groups.

### 3.5 Pharmacokinetics

This sub‐study included a total 30 evaluable subjects.

The geometric least squares mean of biosimilar formulation (*T*) and reference formulation (*R*) and its ratio (*T*/*R*)% obtained from the analysis of ln‐transformed parameters *C*_max_ and AUC_0–*t*_ are summarized in the following (Table 4).

**TABLE 4:** Geometric least squares mean of biosimilar and reference formulations.

Parameters (units)	Proposed biosimilar teriparatide (*T*) (*n =* 15)	Reference teriparatide (*R*) (*n =* 15)	*T*/*R* (%)	*p* ^a^
*C*_max_ in (pg/mL)	108.79	105.89	102.74	0.876
AUC_(0–*t*)_ in min	6132.45	6387.59	96.01	0.865

^a^ Proposed biosimilar teriparatide versus reference teriparatide.

## 4 Discussion

Recombinant human teriparatide is the biologically active N terminal 34‐amino acid fragment of the full‐length, 84‐amino acid native parathyroid hormone [PTH (1–84)]. Teriparatide is the only anabolic agent currently available in India for the treatment of postmenopausal osteoporosis. Unlike antiresorptive agents that prevent bone resorption, teriparatide stimulates bone formation [21]. According to the American Association of Clinical Endocrinologists and the American College of Endocrinology (AACE/ACE) guidelines, teriparatide is the treatment of choice for postmenopausal osteoporosis in patients at high risk of fractures or those who have failed to respond to or cannot tolerate other osteoporosis therapies [22].

This phase 3 study evaluated the similarity between the proposed biosimilar teriparatide and the reference teriparatide in terms of efficacy, safety, pharmacodynamics, and pharmacokinetics in patients with postmenopausal osteoporosis over a 48‐week treatment period. The findings demonstrated a percentage increase in lumbar spine BMD of 8.46 ± 0.94 (mean ± SE) and in femoral neck BMD of 4.03 ± 0.96 (mean ± SE) at the end of 48 weeks (approximately 1 year) in the biosimilar teriparatide group (20 μg daily). These results align with the pivotal Fracture Prevention Trial, where the percentage increase in BMD at the lumbar spine was reported as 9.7 ± 7.4 (mean ± SE) and at the femoral neck as 2.8 ± 5.7 (mean ± SE) after 2 years of treatment with 20 μg teriparatide daily [12].

Similarly, a study on proposed biosimilar teriparatide approved in Japan demonstrated that the percent change from baseline to 52 weeks in lumbar spine (L2–L4) BMD (mean ± SE) was 8.94% ± 6.19% in the RGB‐10 group and 9.65% ± 6.22% in the reference teriparatide group [23].

Bone biomarkers are recognized as reliable indicators of a drug's effect on the rate of future fractures [24]. Among these, serum P1NP has been identified as the most sensitive marker for detecting changes in bone turnover in response to teriparatide therapy [25]. In the current study, both teriparatide preparations significantly improved the bone turnover marker, P1NP, from baseline to Weeks 12, 24, and 48.

The most common treatment‐emergent adverse events (TEAEs) reported in the proposed biosimilar teriparatide group included hyperuricemia (7.0%) and pyrexia (5.3%). In comparison, dizziness (9%) and leg cramps (3%) were the frequently reported TEAEs associated with 20‐μg teriparatide in the Fracture Prevention Trial [23]. Notably, hyperuricemia (7.0%) was the most prevalent TEAE in the biosimilar group, whereas pyrexia (7.9%) was reported at a higher frequency in the reference group.

There are few limitations of the study. Firstly, the study was not designed to compare the effects of teriparatide preparations in the reduction of fractures. However, BMD is well‐established surrogate marker of fractures [26]. Secondly, this study evaluated the impact of teriparatide treatment on BMD only for 1 year.

In conclusion, the proposed biosimilar teriparatide significantly improved lumbar spine and femoral neck BMD at the end of 48 weeks, demonstrating efficacy comparable to the reference teriparatide, with no major safety concerns observed.

## 5 Conclusion

In conclusion, based on the efficacy and safety analysis, the study established noninferiority, along with comparable pharmacokinetics and immunogenicity between Alkem's biosimilar teriparatide and reference teriparatide in patients with postmenopausal osteoporosis.

## Author Contributions

Nitin Kapoor, Thomas Paul, Rajeshwar Nath Srivastava, Saurabh Singh, Sunil Maheshwari, Sushil H. Mankar, Joe Joseph Cherian, Surabhi Maheshwari, and Sudeepti Srivastava have contributed to protocol development, patient recruitment, CSR finalization, and manuscript preparation. Vishal Patil has contributed to patient recruitment and CSR finalization. Awadhesh Kumar Yadav has contributed to patient recruitment, CSR finalization, and manuscript preparation. Girish Bhatia has contributed to patient recruitment and manuscript preparation. Dattatray Pawar, Roshan Pawar, Amol Aiwale, Amitrajit Pal, Yogesh Rane, Vinayaka Shahavi, and Akhilesh Sharma have contributed to protocol development, CSR, and manuscript finalization.

## Disclosure

Code availability: Study ID: ALK05‐TERI1.

Disclaimer: The funding organization has served as study sponsor and has been involved in the design, conduct of the study, in the collection, management, analysis, and interpretation of the data, the preparation, review, and publishing of the manuscript.

## Ethics Statement

This phase III clinical trial has been approved by DCGI (Drugs Controller General of India) and Institutional Ethics Committees and has been performed in accordance with the ethical standards as laid down in the 1964 Declaration of Helsinki, ICH GCP, and its later amendments or comparable ethical standards.

## Consent

Informed consent was obtained from all the individual participants included in the study.

## Conflicts of Interest

The authors declare no conflicts of interest.

## Data Availability

Data are available from the Teriparatide Osteoporosis Study data steering committee upon reasonable request. Please direct inquiries to the corresponding author.
